# Real-time analysis application for identifying bursty local areas related to emergency topics

**DOI:** 10.1186/s40064-015-0817-x

**Published:** 2015-04-03

**Authors:** Tatsuhiro Sakai, Keiichi Tamura

**Affiliations:** Graduate School of Information Sciences, Hiroshima City University, 3-4-1, Ozuka-Higashi, Asa-Minami-Ku, 731-3194 Hiroshima Japan

**Keywords:** Spatiotemporal clustering, Density-based clustering, Social media, Naive Bayes, Burst detection

## Abstract

Since social media started getting more attention from users on the Internet, social media has been one of the most important information source in the world. Especially, with the increasing popularity of social media, data posted on social media sites are rapidly becoming collective intelligence, which is a term used to refer to new media that is displacing traditional media. In this paper, we focus on geotagged tweets on the Twitter site. These geotagged tweets are referred to as georeferenced documents because they include not only a short text message, but also the documents’ posting time and location. Many researchers have been tackling the development of new data mining techniques for georeferenced documents to identify and analyze emergency topics, such as natural disasters, weather, diseases, and other incidents. In particular, the utilization of geotagged tweets to identify and analyze natural disasters has received much attention from administrative agencies recently because some case studies have achieved compelling results. In this paper, we propose a novel real-time analysis application for identifying bursty local areas related to emergency topics. The aim of our new application is to provide new platforms that can identify and analyze the localities of emergency topics. The proposed application is composed of three core computational intelligence techniques: the Naive Bayes classifier technique, the spatiotemporal clustering technique, and the burst detection technique. Moreover, we have implemented two types of application interface: a Web application interface and an android application interface. To evaluate the proposed application, we have implemented a real-time weather observation system embedded the proposed application. we used actual crawling geotagged tweets posted on the Twitter site. The weather observation system successfully detected bursty local areas related to observed emergency weather topics.

**Electronic supplementary material:**

The online version of this article (doi:10.1186/s40064-015-0817-x) contains supplementary material, which is available to authorized users.

**Electronic supplementary material:**

The online version of this article (doi:10.1186/s40064-015-0817-x) contains supplementary material, which is available to authorized users.

## Introduction

In recent years, social media has played a significant role as an alternative source of information (Kavanaugh et al. 2011; Yin et al. 2012). In particular, people actively transmit and collect information about emergency topics, such as natural disasters, weather, diseases, and other incidents (Miyabe et al. 2012; Vieweg et al. 2010). Enhancement of the utilization of social media for emergency management is one of the most interesting issues being discussed in public and governmental institutions. Therefore, a significant number of researchers have focused on the development of emergency topic and event detection via social media. This trend provides an opportunity for addressing new challenges in many different application domains: how to detect where emergency topics occur and what they are going on.

In this study, we focus on geotagged tweets posted on the Twitter site. These geotagged tweets are referred to as georeferenced documents because they usually include not only a short text message, but also the documents’ posting time and location. Users on the Twitter site are referred to as social sensors and geotagged tweets as sensor data observed by the social sensors. Some of studies that focused on these geotagged tweets are as follows. Sakaki et al. (2010) focused on tweets regarding typhoons and earthquakes using associated geographic information to estimate typhoon trajectories and earthquake epicenters using dense regions. Ozdikis et al. (2013) also proposed a method that estimated the geographical location of events in the case of earthquakes reported on Twitter.

It is of value to people interested in a certain topic to observe dense areas where many georeferenced documents related to the topic are located. In this paper, these dense areas are referred to as bursty local areas related to the topic. For example, Tamura and Ichimura (2013) proposed a novel density-based spatiotemporal clustering algorithm that can extract spatially and temporally separated spatial clusters in georeferenced documents. They proposed the (*ε*,*τ*)-density-based spatiotemporal clustering algorithm; the experimental results indicate that their proposed algorithm can extract bursty local areas in a set of geotagged tweets, including keywords related to a topic related to weather topics.

In this paper, we propose a novel real-time analysis application for identifying bursty local areas related to emergency topics. The aim of our new application is to provide new platforms that can identify and analyze the localities of emergency topics. The proposed application is composed of three core computational intelligence techniques: the Naive Bayes classifier technique, the spatiotemporal clustering technique, and the burst detection technique. The (*ε*,*τ*)-density-based spatiotemporal clustering algorithm is a useful algorithm for extracting bursty local areas; however, two functional issues remain unresolved. One issue is that the (*ε*,*τ*)-density-based spatiotemporal clustering algorithm does not support real-time extraction. In Tamura and Ichimura (2013), only a batch clustering algorithm was proposed. The second issue is that the proposed algorithm is based on keywords. Therefore, relevant georeferenced documents are extracted if they include an observed keyword, not an observed topic; and this causes error extraction.

The main characteristics of our application are as follows.

- To extract georeferenced documents including emergency topics as relevant georeferenced documents, our application utilizes the Naive Bayes classifier Manning et al. (2008) technique. As compared to that proposed in Tamura and Ichimura (2013), the proposed application can detect spatiotemporal clusters as bursty local areas with more sensitivity. For example, suppose that there are two sentences, “I saw a snow monkey” and “It is snowing heavily”. These two sentences include a keyword “snow”. The first one is not related to weather topic “snow”. Our application is topic-based; therefore the first one is not extracted as a relevant georeferenced document.
- To identify (*ε*,*τ*)-density-based spatiotemporal clusters in real time, an incremental algorithm for (*ε*,*τ*)-density-based spatiotemporal clustering is used. The target topics of this study are emergency topics; therefore we need to identify local bursty areas in real time. In Tamura and Ichimura (2013), only a batch clustering algorithm was proposed. In contrast, the incremental algorithm extracts (*ε*,*τ*)-density-based spatiotemporal clusters on the arrival of each relevant georeferenced document.
- To identify the burstiness of extracted bursty local areas, we integrate location-based burst detection techniques Tamura and Kitakami (2013) in the proposed application. Burst Kleinberg (2002) is one of the simplest but most effective criterion to measure how hot topics are. The traditional time-based burst detection does not work well for this study because the emergency topics appear in local area. The location-based burst detection techniques can detect the localities of burstiness of emergency topics.

The rest of this paper is organized as follows. In Section Related work, we briefly describe related work. In Section (*ε*,*τ*)-Density-based spatiotemporal clustering, the density-based clustering algorithm and the (*ε*,*τ*)-density-based spatiotemporal clustering algorithm are reviewed. In Section The proposed application, we propose our application and describe the details of the core intelligent computational techniques implemented in it. In Section Experimental result, the experiments for the evaluation of the proposed application is reported. In Section Conclusion, we conclude this paper.

## Related work

In the era of big data, we are witnessing the rapid growth of a new type of information source. Social media has been noticed by a significant number of people; today, we obtain information instantaneously about emergency topics that surround us. In particular, tweets from one of the most widely used micro-blogging services have been involved in many different application domains Java et al. (2007). Tweets include not only rubbish messages transmitted between users, but also comments about and content for social topics and events Jansen et al. (2009). Twitter users are referred to as sensors observing the world, and their tweets as sensor data.

Recently, a huge amount of geotagged tweets are posted because of the popularity of geomobile applications on smartphones. These geotagged tweets are useful for extracting local topics and events (Abdelhaq et al. 2013; Hiruta et al. 2012; Hong et al. 2012; Musleh 2014). In particular, geotagged tweet have been expected to be utilized for analyzing emergency topics like natural disasters (Mandel et al. 2012) and epidemic (Hwang et al. 2013).

The most related work is Aramaki et al. (2011) which were conducted by Aramaki et al. They proposed a novel method for detecting influenza epidemics using tweets; their method utilized classifiers, such as support vector machine (SVM) and Naive Bayes to extract the tweets that included topics about influenza. Their geocoding technique was used to map each tweet to a region in Japan. Moreover, their proposed system visualized the increase and decrease in the number of related tweets in each region. As indicated above, their approach is more closely related to our work than any other; however, the method cannot identify bursty areas in detail. Our proposed method can detect large regions like prefectures.

Murakami et al. analyzed tweets about the 2011 Tohoku earthquake and tsunami Murakami and Nasukawa (2012). They presented the analysis results of social media data using text mining tools. Marcus et al. (2011) developed a visualizing system called Twitinfo. Their system is suitable for analyzing global topics and their time change. Moreover, the system visualized the sentiment of each topic. Karimi et al. (2013) proposed classifying methods that can identify high-value tweets related to disasters. Their method showed a good performance; however, they proposed only the classifying methods.

Kaneko et al. (2013) proposed a novel method for detecting images of events such as the cherry-blossom festival using tweets that included geo-tags and images. First, their method checks the number of tweets posted on a day and the preceding day and also extracts keywords from the posts. Next, it searches and collects images of tweets using these keywords. Moreover, their method performs clustering using the feature value of the collected images and selects a representative image as an event; however, their method cannot detect bursty areas in detail, because it checks only the changes from one day to the next.

Typhoon Real Time Watcher (2014) was developed recently. This system can observe and track social typhoon reports about their rain, wind, and damages, which are provided by a weather forecast company. This system utilized social reports manually selected; therefore, the reliability is assured. The system is good tool for analyzing typhoons; however, it is difficult to obtain real-time situation because it utilizes only social typhoon reports provided by a weather forecast company. NTT DoCoMo proposed an application called Geographical TimeLine that uses its own auto GPS system and Twitter. The application can detect in real time a place where many people are using its auto GPS system. Moreover, the application can analyze events that occur by exploiting Twitter; however, it requires an auto GPS system.

## (*ε*,*τ*)-Density-based spatiotemporal clustering

This section reviews the density-based clustering framework and the (*ε*,*τ*)-density-based spatiotemporal clustering algorithm proposed in Tamura and Ichimura (2013). The (*ε*,*τ*)-density-based spatiotemporal clustering algorithm is the improved version of DBSCAN algorithm Ester et al. (1996).

### Density-based spatiotemporal criteria

The (*ε*,*τ*)-density-based spatiotemporal clustering algorithm is based on the density-based spatial clustering algorithm Sander et al. (1998). In the density-based spatial clustering algorithm, spatial clusters are dense areas that are separated from areas of lower density. In other words, areas with high densities of data points can be considered spatial clusters, whereas those with low density cannot. The key concept underpinning the use of the density-based spatial clustering algorithm indicates that, for each data point within a spatial cluster, the neighborhood of a user-defined radius must contain at least a minimum number of points; that is, the density in the neighborhood must exceed some predefined threshold.

The algorithm that has affected the density-based spatial clustering algorithm is the DBSCAN algorithm, which was first introduced by Ester et al. (1996). The (*ε*,*τ*)-density-based spatiotemporal clustering algorithm Tamura and Ichimura (2013) is a natural extension of DBSCAN. DBSCAN utilizes *ε*-neighborhood density and recognizes areas in which densities are higher than in other areas. However, it does not consider temporal changes. By contrast, the (*ε*,*τ*)-density-based spatiotemporal clustering algorithm extracts (*ε*,*τ*)-density-based spatiotemporal clusters that are both temporally and spatially-separated from other spatial clusters.

In the (*ε*,*τ*)-density-based spatiotemporal clustering algorithm, areas with high spatial-/temporal-densities of data points can be considered spatiotemporal clusters, whereas those with low spatial-/temporal-densities cannot. To extract spatiotemporal clusters, we extend the definitions of density in DBSCAN. Suppose that a emergency topic is getting more attention in a local area. Users in the local area starts posting geotagged tweets related to the emergency topic; therefore, if we detect high dense regions in which there are many geotagged tweets, we can analyze local bursty areas where emergency topic is getting more attention from local people.

### Definitions

The (*ε*,*τ*)-density-based neighborhood, which indicates the density of the neighborhood of a georeferenced document, is defined as follows.

#### Definition 1 ((*ε*,*τ*)-density-based neighborhood).

The (*ε*,*τ*)-density-based neighborhood of a georeferenced document gdp, which is denoted by *N*_(*ε*,*τ*)_(*gdp*), is defined as

(1) $$\begin{array}{@{}rcl@{}} N_{(\epsilon, \tau)}(gdp) &=& \{gdq \in GD | dist(gdp,gdq) \\&\leq& \epsilon \ and \ iat(gdp,gdq) \leq \tau\}, \end{array} $$

*where the function dist returns the distance between georeferenced documents gdp and gdq, and the function iat returns the interarrival time between them.*

**Figure 1:**
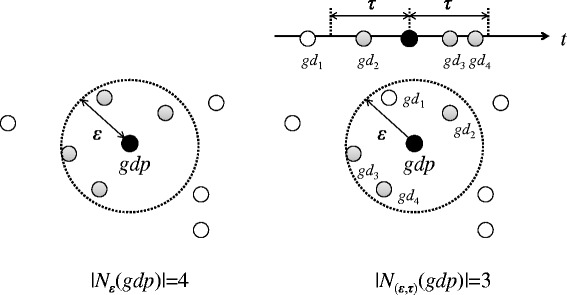
Definition 1 in the right-hand side of Figure 1, there are three documents, *N*_(*ε*,*τ*)_(*g* *d* *p*)={*g* *d*_2_,*g* *d*_3_,*g* *d*_4_}.

Figure 1 shows an example of an (*ε*,*τ*)-density-based neighborhood. In DBSCAN, the neighborhood of document *gdp* is a set of documents that exist within *ε* from *gdp*. In the left-hand side of Figure 1, there are four documents in the neighborhood of *gdp*. Conversely, the (*ε*,*τ*)-density-based neighborhood of *gdp* is a set of documents that exist within *ε* from *gdp*, where each document in the (*ε*,*τ*)-density-based neighborhood is posted in *τ* before or after the posted time of document *gdp*. The right-hand side of Figure 1 shows the example of the (*ε*,*τ*)-density-based neighborhood. In this example, there are three documents, *N*_(*ε*,*τ*)_(*gdp*)={*gd*_2_,*gd*_3_,*gd*_4_}. Document *gd*_1_ is within *ε* from document *gdp*; however, it is not in *N*_(*ε*,*τ*)_(*gdp*), because it is not posted in *τ* before or after the posted time of document *gdp*.

#### Definition 2 (Core and Border).

A georeferenced document *gdp* is called a core georeferenced document if there is at least a minimum number of georeferenced documents, MinGDoc, in the (*ε*,*τ*)-density-based neighborhood *N*_(*ε*,*τ*)_(*dp*) of that georeferenced document (|*N*_(*ε*,*τ*)_(*gdp*)|≥*MinGDoc*). Otherwise, (|*N*_(*ε*,*τ*)_(*gdp*)|≤*MinGDoc*), a georeferenced document gdp is called a border georeferenced document.

**Figure 2:**
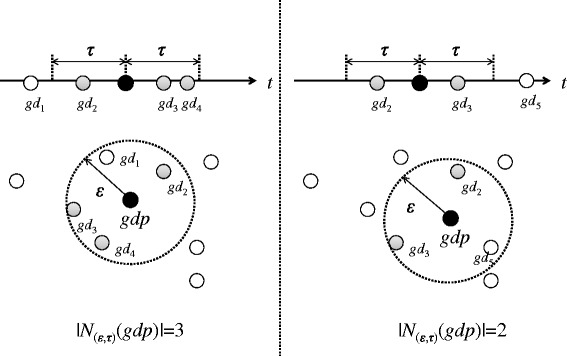
This figure shows an example of Definition 2 and Definition 3. Suppose that *MinGdoc* is three. In the left-hand side of Figure 2, *gdp* is a core georeferenced document. In the right-hand side of Figure 2, *gdp* is not a core georeferenced document.

Suppose that *MinGDoc* is set to three. In the left-hand side of Figure 2, *gdp* is a core georeferenced document, because |*N*_(*ε*,*τ*)_(*gdp*)| is three. In the right-hand side of Figure 2, *gdp* is not a core georeferenced document, because |*N*_(*ε*,*τ*)_(*gdp*)| is three.

#### Definition 3 ((*ε*,*τ*)-density-based directly reachable).

Suppose that the georeferenced document *gdq* is in the (*ε*,*τ*)-density-based neighborhood of georeferenced document gdp. If the number of georeferenced documents in the (*ε*,*τ*)-density-based neighborhood of georeferenced document gdp is greater than or equal to MinGDoc, i.e., is |*N*_(*ε*,*τ*)_(*gdp*)|≥*MinGDoc*, document gdq is (*ε*,*τ*)-density-based directly reachable from gdp. In other words, georeferenced documents in the (*ε*,*τ*)-density-based neighborhood of a core georeferenced document are (*ε*,*τ*)-density-based directly reachable from the core georeferenced document.

On the left-hand side of Figure 2, *gdp* is a core georeferenced document, because *N*_(*ε*,*τ*)_(*dp*)≥*MinGDoc*. Then, *gd*_1_, *gd*_2_ and *gd*_4_ are in the (*ε*,*τ*)-density-based neighborhood of document *gdp*. These three georeferenced documents are (*ε*,*τ*)-density-based directly reachable from *gdp*.

#### Definition 4 ((*ε*,*τ*)-density-based reachable).

Suppose that there is a georeferenced document sequence (*gdp*_1_,*gdp*_2_,⋯,*gdp*_*n*_) and the *i*+1-th georeferenced document *gdp*_*i*+1_ is (*ε*,*τ*)-density-based directly reachable from the i-th georeferenced document *gdp*_*i*_. Georeferenced document *gdp*_*n*_ is (*ε*,*τ*)-density-based reachable from georeferenced document *gdp*_1_.

**Figure 3:**
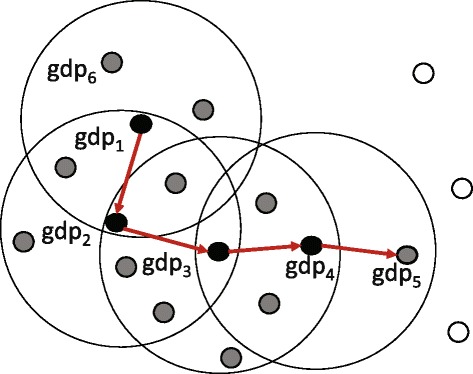
This figure shows an example of Definition 4. Georeferenced document *d* *p*_5_ is (*ε*,*τ*)-density-based reachable from *g* *d* *p*_1_.

Figure 3 shows an example of definition 4. In a georeferenced document sequence (*gdp*_1_,*gdp*_2_,⋯,*gdp*_5_), the *i*+1-th georeferenced document *gdp*_*i*+1_ is (*ε*,*τ*)-density-based directly reachable from *gdp*_*i*_. Therefore, *dp*_5_ is (*ε*,*τ*)-density-based reachable from *gdp*_1_.

#### Definition 5 ((*ε*,*τ*)-density-based connected).

Suppose that georeferenced documents *gdp* and *gdq* are (*ε*,*τ*)-density-based reachable from a georeferenced document *gdo*, which is an arbitrary georeferenced document. If |*N*_(*ε*,*τ*)_(*gdo*)|≥*MinGDoc*, we denote that *gdp* is (*ε*,*τ*)-density-based connected to *gdq*.

**Figure 4:**
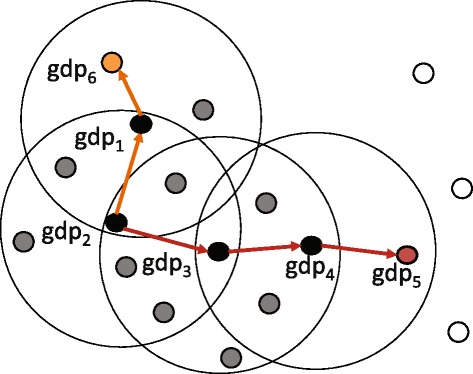
This figure shows an example of Definition 5. Georeferenced document *g* *d* *p*_6_ is (*ε*,*τ*)-density-based connected to *g* *d* *p*_5_.

Figure 4 shows an example of definition 5. In this example, *gdp*_6_ is (*ε*,*τ*)-density-based connected to *gdp*_5_, because *gdp*_5_ is (*ε*,*τ*)-density-based reachable from *gdp*_2_ and *gdp*_6_ is (*ε*,*τ*)-density-based reachable from *gdp*_2_.

### (*ε*,*τ*)-Density-based spatiotemporal cluster

An (*ε*,*τ*)-density-based spatiotemporal cluster consists of two types of georeferenced document: core georeferenced documents, which are mutually (*ε*,*τ*)-density-based reachable; and border georeferenced documents, which are (*ε*,*τ*)-density-based directly reachable from the core georeferenced documents. An (*ε*,*τ*)-density-based spatiotemporal cluster is defined as follows.

An (*ε*,*τ*)-density-based spatiotemporal cluster (*DSC*) in a georeferenced document set *GD* satisfies the following restrictions: ***(1)*** ∀*gdp*, *gdq*∈*GD*, if and only if *gdp*∈*DSC* and *gdq* is (*ε*,*τ*)-density-based reachable from *gdp*, and *gdq* is also in *DSC*. ***(2)*** ∀*gdp*, *gdq*∈*DSC*, *gdp* is (*ε*,*τ*)-density-based connected to *gdq*.

Even if *gdp* and *gdq* are border georeferenced documents, *gdp* and *gdq* are in a same (*ε*,*τ*)-density-based spatiotemporal cluster if *gdp* is (*ε*,*τ*)-density-based connected to document *gdq*.

### Algorithm

Algorithm 1 describes the batch algorithm for (*ε*,*τ*)-density-based spatiotemporal clustering. In this algorithm, for each georeferenced document *gdp* in *GD*, the function **IsClustered** checks whether document *gdp* is already assigned to a spatiotemporal cluster. Then, the (*ε*,*τ*)-density-based neighborhood of document *gdp* is obtained using the function **GetNeighborhood**. If georeferenced document *gdp* is a core document according to Definition 2, it is assigned to a new spatiotemporal cluster, and all the neighbors are queued to *Q* for further processing. The processing and assignment of georeferenced documents to the current spatiotemporal cluster continues until the queue is empty. The next georeferenced document is dequeued from queue *Q*. If the dequeued georeferenced document is not already assigned to the current spatiotemporal cluster, it is assigned to the current spatiotemporal cluster. Then, if the dequeued document is a core document, the georeferenced documents in the (*ε*,*τ*)-density-based neighborhood of the dequeued georeferenced document are queued in queue *Q* using the function **EnNniqueQueue**, which places the input georeferenced documents into queue *Q* if they are not already in queue *Q*.



## The proposed application

In this section, we propose a novel real-time analysis application for identifying bursty local areas related to emergency topics. A system overview and the system process are presented.

### Aim

The aim of developing our application is to provide a platform that can analyze bursty local areas related to emergency topics in real time. Suppose that it is raining in area “A”. As the rain becomes heavily in area “A”, geotagged tweets related to the topic “rain” may be posted in the area. At the same time, the density of posted geotagged tweets become greater than usual. If the density areas are extracted, we can detect bursty local areas related to the emergency topic.

### System overview

**Figure 5:**
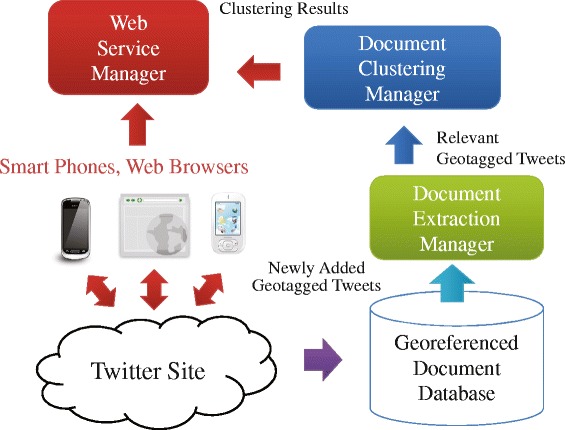
System overview of the proposed application. In the system, the application server has three main managers: *Document* *Extraction* *Manager*, *Document* *Clustering* *Manager*, and *Web* *Service* *Manager*. A georeferenced document database is constructed on the application server. We can observe bursty local areas of emergency topics through a Web application interface and an Android application interface.

Figure 5 shows an overview of the system for the proposed application. In the system, the application server has three main managers: *DocumentExtractionManager*, *DocumentClusteringManager*, and *WebServiceManager*. We can observe bursty local areas of emergency topics through a Web application and an Android application.

Our system has a georeferenced document database that contains geotagged tweets crawled by the Twitter site. Let *gd*_*i*_ denote the *i*-th georeferenced document in *GDS*={*gd*_1_,⋯,*gd*_*n*_}; then, *gd*_*i*_ consists of three items: *gd*_*i*_=<*text*_*i*_,*pt*_*i*_,*pl*_*i*_ >, where *text*_*i*_ is the content (e.g., title, short text message, and tags), *pt*_*i*_, which is the time when the georeferenced document was posted, and *pl*_*i*_, which is the location where *gd*_*i*_ was posted or is located (e.g., latitude and longitude).

Each georeferenced document arrives step by step. The following steps are executed on the application server.

1. *DocumentExtractionManager* fetches a georeferenced document, which is newly inserted in the georeferenced document database.
2. *DocumentExtractionManager* classifies the fetched georeferenced document *gd*_*i*_ using a Naive Bayes classifier. If and only if *gd*_*i*_ is classified to “positive” class, which means *gd*_*i*_ is related to an observed emergency topic, go to the next step.
3. *DocumentClusteringManager* executes the incremental algorithm for extracting (*ε*,*τ*)-density-based spatiotemporal clustering, for which there are two input data: *gd*_*i*_ and a set of current extracted (*ε*,*τ*)-density-based spatiotemporal clusters.
4. For each (*ε*,*τ*)-density-based spatiotemporal cluster, the burstiness of the cluster is calculated.
5. *WebServiceManager* provides Web-based application interfaces to access information about extracted bursty local areas.

Our system is not dependence on any language. For, example, the Naive Bays classifier can be conducted if any morphological analysis tool is provided. Moreover, the density-based spatiotemporal algorithm is without dependence on language. Therefore, we can extend the proposed system to another language if we modified the Web-based Interfaces and the Android applications in the language.

### Naive Bayes classifier

The proposed application requires that georeferenced documents related to an observed emergency topic are extracted. Georeferenced documents including the observed emergency topic contain many kinds of keyword. Therefore, a keyword-based search is not effective for extraction. For example, suppose that an observed emergency topic is “rain”. Sequences “It is raining” and “It could rain this weekend” include the keyword “rain”; but, they have different topics. In this case, only “It is raining” is extracted as a relevant georeferenced document related to the topic “rain”.

To satisfy this requirement, in *DocumentExtractionManager*, the Naive Bayes classifier technique is utilized in order to extract georeferenced documents. A Naive Bayes classifier is a simple probabilistic classifier based on applying Bayes’ theorem, which is based Bayesian statistics with naive independence assumptions. *DocumentExtractionManager* classifies geotagged tweets as either “positive” class or “negative” class manually, where “positive” class is related to the observed emergency topic and “negative” class is not. Georeferenced documents in the “positive” class are the relevant georeferenced documents.

In this study, the Naive Bayes classifier is based on keywords in text data included in georeferenced documents. Let *CLASS*={*positive*,*negative*} be a set of classes. The posterior probability that is the georeferenced document *gd* belongs the class *class*∈*CLASS* is

(2) $${} P(class|gd)=\frac{P(class)P(gd|class)}{P(gd)} \propto {P(class)P(gd|class)},  $$

where *P*(*class*) is the prior probability of *class* and *P*(*gd*|*class*) is the likelihood.

The Naive Bayes classifier requires a training data set including multiple georeferenced documents that are classified in one class in *CLASS*. Let a training data set *TDS* be *TDS*={(*tgd*_1_,*c*_1_),(*tgd*_2_,*c*_2_),⋯,(*tgd*_*m*_,*c*_*m*_)}, where *c*_*i*_∈*CLASS*. A set of all words in *class* is denoted by *W*_*class*_={*word*_1_,*word*_2_,⋯,*word*_*l*_}.

The georeferenced *gd* represents a bag-of-words *word*_1_,*word*_2_,⋯,*word*_*numw*(*gd*)_, where let *numw*(*gd*) be the number of words in *gd*.

(3) $$\begin{array}{@{}rcl@{}} P(gd|class) &=& P({word}_{1} \wedge {word}_{2} \wedge \cdots \wedge {word}_{k}|class)\\ &=& \prod_{i}^{numw(gd)} P({word}_{i} | class) \end{array} $$

where, *P*(*word*_*i*_|*class*) is the probability that *word*_*i*_ occurs in *class*. *P*(*word*_*i*_|*class*) is defined as

(4) $$\begin{array}{@{}rcl@{}} P({word}_{i}|class) = \frac{OW({word}_{i}, class)+1}{\sum_{j=1}^{|W_{class}|}(OW({word}_{j}, class)+1)}, \end{array} $$

where *OW*(*word*_*i*_,*class*) is the number of occurrence of *word*_*i*_ in *class*.

The assigned class of *gd* denoted by *c*_*gd*_∈*CLASS* is determined by finding the maximum posterior probability.

(5) $$\begin{array}{@{}rcl@{}} c_{gd} &=& \arg \max_{class} P(class|gd)  \\ &=& \arg \max_{class} \left(P(class) \prod_{i}^{numw(gd)} P({word}_{i} | class) \right)  \\ \end{array} $$

### Incremental algorithm

In the incremental algorithm, the algorithm updates the states of the extracted spatiotemporal clusters and extracts new spatiotemporal clusters every time a georeferenced document is added. Algorithm 2 describes the incremental (*ε*,*τ*)-density-based spatiotemporal clustering algorithm, which extracts (*ε*,*τ*)-density-based spatiotemporal clusters based on every georeferenced document that arrives for real-time extraction. There are two features in the incremental (*ε*,*τ*)-density-based spatiotemporal clustering algorithm: limited re-clustering and merging.



When a georeferenced document is added to the georeferenced documents, existing (*ε*,*τ*)-density-based spatiotemporal clusters must be updated; but the added georeferenced document affects only its (*ε*,*τ*)-density-based neighborhood within *τ* directly. Function **GetRecentData**(*gd*) returns *gd*’s (*ε*,*τ*)-density-based neighborhood within *τ*. After the (*ε*,*τ*)-density-based neighborhood is extracted to generate seeds and these seed georeferenced documents are re-clustered again.

In the incremental algorithm, during re-clustering, some (*ε*,*τ*)-density-based spatiotemporal clusters need to be appended to other (*ε*,*τ*)-density-based spatiotemporal clusters. Suppose that (*ε*,*τ*)-density-based spatiotemporal cluster *stc* is expanding. If a core georeferenced document in *stc* includes a georeferenced document, which is clustered in *stc*^′^, *stc*^′^ is appended to *stc*. Function **AppendClusters** appends two spatiotemporal clusters and return a appended spatiotemporal cluster.

### Burst detection

To identify the burstiness of extracted areas, we integrate location-based burst detection techniques Tamura and Kitakami (2013) in the proposed algorithm. We extended the location-based burst detection algorithm for detecting the burstiness of (*ε*,*τ*)-density-based spatiotemporal clusters. In the location-based burst detection algorithm, there are two sequences, that of the number of georeferenced documents and that of the number of relevant georeferenced documents.

In this study, a sequence of the number of all the relevant georeferenced documents is referred to as the sequence of georeferenced documents and a sequence of the number of relevant georeferenced documents in each (*ε*,*τ*)-density-based spatiotemporal cluster is referred to as the sequence of relevant georeferenced documents. Moreover, the influence rates of georeferenced documents gradually decrease according to distance from the center of each cluster.

### Application interfaces

Two application interfaces are used for accessing information on bursty local areas related to emergency topics: a Web application interface and an android application interface. These two types of application interfaces access a application server and users can analyze bursty local areas through the interfaces. There is a geographical map on the Web application interface and the android application interface. Bursty local areas mapped on the geographical map, and each geotagged tweets in extracted spatiotemporal clusters are shown in the geographical map. Moreover, we can watch posted image with geotagged tweets.

We implemented a real-time weather observation system embedded with the proposed application. The real-time weather observation system provides the functions that show bursty local areas in which the weather is receiving more attention from many people located in these local areas. We can observe how observed weather is going on in bursty local areas through this system. The weather, such as, rain, snow, and wind cause a severe natural disaster; therefore, this system contributes an analysis of the weather forecast.

**Figure 6:**
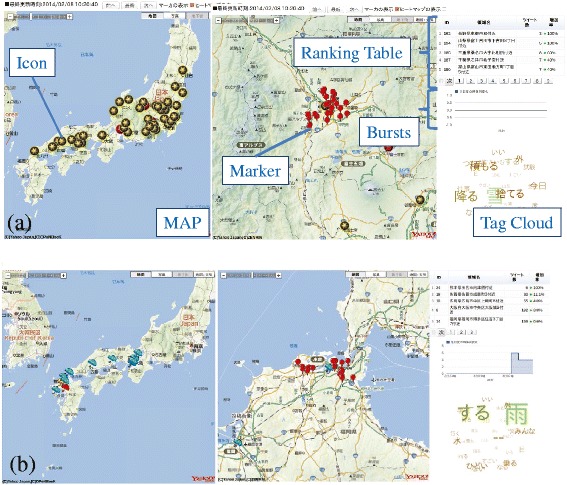
Screen shots of Web application interface.**(a)** shows screen shots of the “snow” observation application that we have implemented, **(b)** shows screen shots of the “rain” observation application that we have implemented. The Web application interface consists of four components: a map, a ranking table, a chart of bursts, and tag cloud. Icons, which indicate extracted bursty areas, are mapped on the map. If users click an icon, markers, which represents geotagged tweets located in the extracted bursty area are appeared. If the users click each marker, a window including the text data of geotagged tweet is opened. The ranking table is a ranking list of extracted bursty areas. Extracted bursty area are ranked by increasing rate of the number of geotagged tweets. An additional movie file shows this in more detail [see Additional file 1].

**Figure 7:**
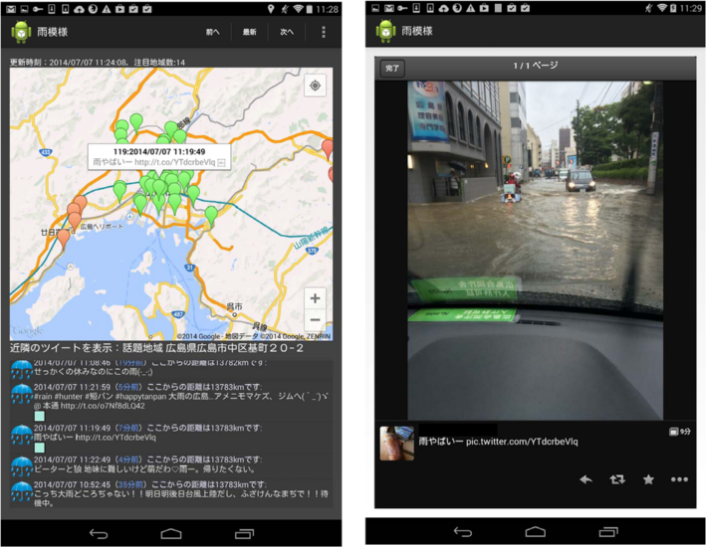
Screen shots of android application interface. This Figure shows the screen shots of the Android application. These screen shots shows the extracted bursty area in Hiroshima on July 7, 2014. The left-side of the figure shows map with extracted clusters. The right-side of the figure shows a photo including in a tweet in an extracted tweets. The tweet says “A heavy rain!". Moreover, the user who posted this tweet posted the photo that shows a flood on the load. An additional movie file shows this in more detail [see Additional file 2].

Figures 6 and 7 show screen shots of the Web application interface and the Android application interface to the real-time weather observation system, which are implemented by us. Figure 6 (a) shows screen shots on February 8, 2014. It snowed heavily in Japan; especially, the Tokyo metropolitan region and Koshin region had heavy snow on February 8, 2014. The icons of snow crystal indicates extracted bursty local areas. Through the system, we can know what weather is going on in Japan. Figure 6 (b) shows screen shots on July 3, 2014. It was rainy in western Japan. The icons of umbrella indicates extracted bursty local areas. If we click or touch these icons, we can observed the details of the selected bursty local areas. An additional movie file shows this in more detail [see Additional file 1].

Figure 7 shows the screen shots of the Android application interface to the real-time weather observation system. These screen shots shows the extracted bursty area in Hiroshima on July 7, 2014. On July 7, it rained heavily in Hiroshima. The photo in the screen shot was posted only 5 minutes ago when the screen was captured. In the center of Hiroshima, we observed that flood damage occurred through the posted photo. An additional movie file shows this in more detail [see Additional file 2]. Table 1 shows that Precipitation on July 7, 2014 in Hiroshima. This data is provided by Japan Meteorological Agency. Table 1 indicates that it rained heavily in Hiroshima at 11:00. This is in consistency with this Figure.

**Table 1:** **Precipitation on July 7, 2014 in Hiroshima**

**Time**	6	7	8	9	10	11	12	13	14	15	16	17	18
**Precipitation (mm)**	0.5	0.5	1.5	20.5	3.0	30.5	10.5	7.5	6.0	3.5	2.5	0.0	0.0

## Experimental result

To evaluate the proposed application, we implemented real-time weather observation system. In our experiments, we used geotagged tweets obtained by crawling Twitter posts and extracted bursty areas in real time. We collected geotagged tweets from the Twitter site using its Streaming API. In the experiments, we observed two topics “snow” and “rain”. We observed each topic in one of two periods. During January and February 2014, we evaluated whether the proposed application can identify bursty areas according to the topic “snow”. In particular, we focused on 12 days in these two months on which it snowed heavily. During the second period, which comprised June and July 2014, we evaluated whether the proposed application can identify bursty areas according to the topic “rain”. In particular, we focused on 16 days in these two months on which it rained heavily.

In the experiments, we evaluate the performance of the Naive Bayes classifier using cross-validation and the extraction rates of the topics “snow” and “rain” in Japan. Moreover, we observed in real time to clearly the availability of the proposed application. The parameters in the experiment were set as : *ε*=5 *km*, *τ*=3600 *s*, and *MinGDoc* was 3. We conducted several experiments and we select the best parameters.

### Cross-validation

Two training data sets *TDS* for “snow” and “rain” were composed of 2,500 geotagged tweets. One consisted of geotagged tweets that included “snow” as a keyword that were posted on February 8. The other consisted of geotagged tweets that included “rain” as a keyword that were posted on June 4. In these two train data sets, the geotagged tweets in the *TDS* were labeled manually. The number of geotagged tweets in the “positive” class, which means including topic “snow”, and the “negative” class was 1648 and 852, respectively. Moreover, the number of geotagged tweets in the “positive” class, which means including the topic “rain”, and the “negative” class was 897 and 1603, respectively.

**Figure 8:**
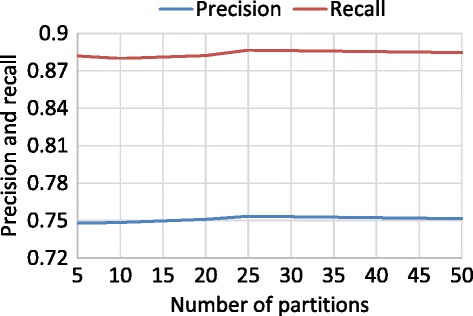
Cross-validation of “snow”.

**Figure 9:**
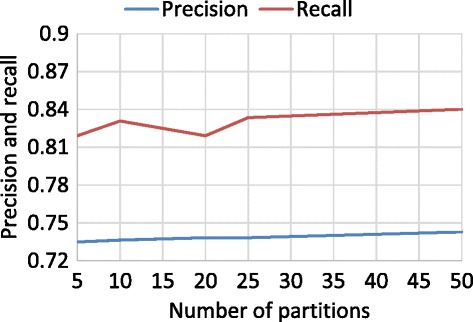
Cross-validation of “rain”.

To evaluate the Naive Bayes classifier, we performed a cross-validation. The number of partitions for the cross-validation was 5, 10, 20, 25, and 50. Figures 8 and 9 show the recall and precision values for each number of partitions for “snow” and “rain”, respectively. The range of recall is from 87% to 89% and from 81% to 84% for “snow” and “rain”, respectively. The range of precision is from 74% to 76% and from 73% to 74% for “snow” and “rain”, respectively.

On July 3, the Naive Bayes classifier extracted 4738 geotagged tweets that included “rain” as a keyword in the “positive” class. We evaluated this classification. The precision of the 4738 geotagged tweets extracted on July 3 is 93.4%. Therefore, according to the observed geotagged tweets using the Naive Bayes classifier, the proposed framework can extract topic-related geotagged tweets with high precision and recall.

### Extraction rates

We collected newspaper articles including the topic “snow” and “rain” and extracted areas heavily snowed and rained that were reported from the articles. The number of extracted areas is shown in Tables 2 and 3. There are 106 and 131 areas that are reported to be a heavily rainy and snowy areas in newspaper from January to February and June to July in 2014, respectively. Tables 2 and 3 show the number of crawled geotagged tweets at each day. The average of the number of geotagged tweets is about 300,000 and 350,000.

**Table 2:** **Data set and detected bursty areas related to topic “snow”**

**Date**	**Number of**	**Number of**	**Number of**	**Number of**	**Detectio n**
	**geotagged**	**extracted**	**heavily**	**detected**	**rates**
	**tweets**	**tweets**	**rainy**	**heavily rainy**	**(** ***B*** **/** ***A*** **× 100)**
			**areas (** ***A*** **)**	**areas (** ***B*** **)**	
2014/1/10	282370	2665	6	3	50.0
2014/1/14	284215	981	1	0	0.00
2014/1/17	283809	995	4	0	0.00
2014/2/6	284065	2821	2	1	50.0
2014/2/8	350867	27823	38	31	81.6
2014/2/10	304380	3424	1	0	0.00
2014/2/11	289628	3564	1	1	100
2014/2/13	306106	3953	1	1	100
2014/2/14	378368	21834	23	17	73.9
2014/2/15	256378	10060	21	15	71.4
2014/2/16	307708	5121	7	2	28.6
2014/2/18	262145	2325	1	1	100

**Table 3:** **Data set and detected bursty areas related to topic “rain”**

**Date**	**Number of**	**Number of**	**Number of**	**Number of**	**Detection**
	**geotagged**	**extracted**	**heavily**	**detected**	**rates**
	**tweets**	**tweets**	**rainy**	**heavily rainy**	**(** ***B*** **/** ***A*** **× 100)**
			**areas (** ***A*** **)**	**areas (** ***B*** **)**	
2014/6/4	325095	2249	14	3	21.4
2014/6/6	312145	6401	19	7	70.0
2014/6/7	330540	4433	6	2	33.3
2014/6/13	346507	2589	4	0	0.00
2014/6/16	340675	750	4	2	50.0
2014/6/22	411863	4172	2	0	0.00
2014/6/23	355384	700	5	3	60.0
2014/6/25	393441	2331	21	11	52.4
2014/6/29	441959	4838	8	5	62.5
2014/7/3	341770	4738	16	6	37.5
2014/7/7	376734	4173	4	3	75.0
2014/7/8	366887	1405	5	4	80.0
2014/7/9	374707	4704	5	2	40.0
2014/7/10	395061	4803	3	0	0.00
2014/7/11	383704	1763	9	3	33.3
2014/7/19	412403	5369	6	3	50.0

As mentioned above, the number of heavily snowy and rainy areas were 106 and 131 respectively. Tables 2 and 3 show number of extracted tweets by using the Naive Bayes classifier, number of heavily snowy and rainy areas (*A*), and number of detected heavily snowy and rainy areas (*B*), sorted by date. The value of (*B*/*A*×100) indicates the detection rates of the identification. The detection rates are less than 50% for 8 out of 12 days and 8 out of 16 days respectively, because there are many country areas that had heavily where no geotagged tweets were posted.

We also evaluated another type of detection rates; Tables 4 and 5 show the detection rates of detected heavily snowy and rainy areas after removing areas with no posted geotagged tweets. Tables 4 and 5 show (*B*/*C*×100) that indicate another detection rates. The detection rates are larger than 50% for 8 out of 12 days and 12 out of 16 days respectively. The average of detection rates are 83.7% and 73.0%. Threfore, areas where there are geotagged tweets, the proposed framework can identifly heavily rainy areas with high detection rates. However, some areas are low detection rates because time interval posted geotagged tweets of those areas are long. Those areas was raining all day on the observed day, for example “Saeki Oita”, “Nobeoka Miyazaki” and “Tsuno Miyazaki” on June 4.

**Table 4:** **Detected bursty areas related to topic “snow” after removing areas with no posted geotagged tweets**

**Date**	**Number of**	**Number of rainy**	**Detection**
	**heavily rainy**	**detected heavily**	**rates**
	**areas (** ***C*** **)**	**areas (** ***B*** **)**	**(** ***B*** **/** ***C*** **×100)**
2014/1/10	3	3	100
2014/1/14	1	0	0.00
2014/1/17	1	0	0.00
2014/2/6	2	1	50.0
2014/2/8	33	31	93.9
2014/2/10	1	0	0.00
2014/2/11	1	1	100
2014/2/13	1	1	100
2014/2/14	20	17	85.0
2014/2/15	19	15	78.9
2014/2/16	5	2	40.0
2014/2/18	1	1	100

**Table 5:** **Detected bursty areas related to topic “rain” after removing areas with no posted geotagged tweets**

**Date**	**Number of**	**Number of**	**Detection**
	**heavily rainy**	**detected**	**rates**
	**areas (** ***C*** **)**	**heavily rainy**	**(** ***B*** **/** ***C*** **×100)**
		**areas (** ***B*** **)**	
2014/6/4	8	3	37.5
2014/6/6	9	7	77.8
2014/6/7	3	2	66.7
2014/6/13	1	0	0.00
2014/6/16	3	2	66.7
2014/6/22	1	0	0.00
2014/6/23	3	3	100
2014/6/25	11	11	100
2014/6/29	6	5	83.3
2014/7/3	11	6	54.5
2014/7/7	4	3	75.0
2014/7/8	4	4	100
2014/7/9	3	2	66.7
2014/7/10	0	0	-
2014/7/11	3	3	100
2014/7/19	4	3	75.0

### Real time extraction

**Figure 10:**
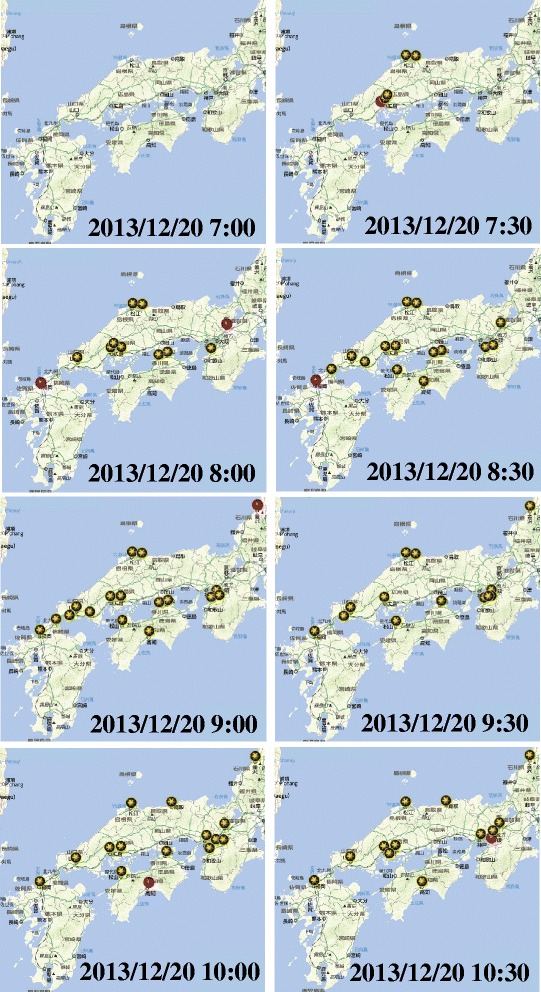
Extracted bursty areas in western Japan on December 20, 2013.

**Figure 11:**
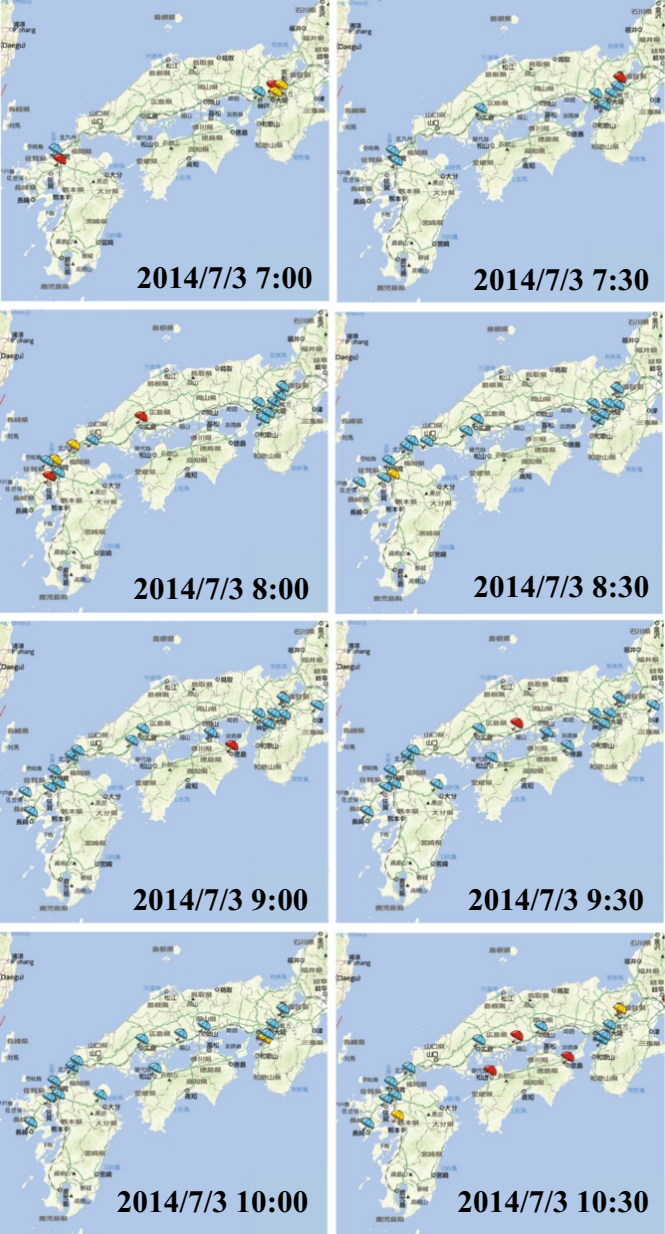
Extracted bursty areas in western Japan on July 3, 2014.

Figure 10 shows that alteration of extracted bursty areas associated with topic “snow” from moment to moment on December 20, 2013. The western part of Japan had first snow in the morning. We observed the system in real time. As the expanding snowfall areas, the number of extracted bursty areas increased. We could analyzed and identify which areas had heavy snowfall and what were tweeting. Figure 11 shows that alteration of extracted bursty areas associated with topic “rain” from moment to moment on July 3, 2014. The western part of Japan had heavy storm in the morning; especially, in the northern part of Kyushu, which is located at the west end of Japan, torrential rainfall occurred. Figure 11 shows that many bursty areas were extracted in the northern part of Kyushu at each time.

**Figure 12:**
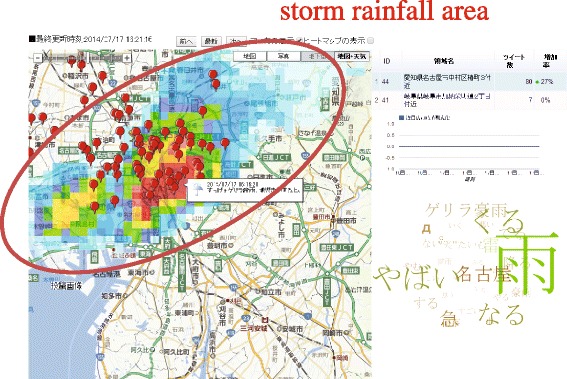
Case study: This figure shows that a bursty local area extracted in Nagoya City, which is located in the center of Japan at 16:21 on July 17, 2014. A sudden heavy rain was observed in Nagoya at that time. The amount of precipitation analyzed by radar-AMeDAS was overloaded on the map. In the tag clouds, there were frequent keywords like, “Sudden”, “Risky”, and “Guerrilla Rainstorm”.

To discuss potential extension of the proposed application, we observed local heavy rain called “Guerrilla Rainstorm”. through the system. Figure 12 shows that a bursty local area extracted in Nagoya City, which is located in the center of Japan at 16:21 on July 17, 2014. A sudden heavy rain was observed in Nagoya at that time. The amount of precipitation analyzed by radar-AMeDAS was overloaded on the map. The system could identify a rainfall area as a local bursty area. In the tag clouds, there were frequent keywords like, “Sudden”, “Risky”, and “Guerrilla Rainstorm” appeared in this area. The tag clouds showed that this area was dangerous because of heavy rainfall.

## Conclusion

In this paper, we proposed a novel real-time analysis application for identifying bursty local areas related emergency topics. The aim of our new application is to provide a new platform that can identify and analyze the localities of emergency topics. Three core computational intelligence techniques are applied in our applications: the Naive Bayes classifier technique, the spatiotemporal clustering technique, and the burst detection technique. Moreover, we developed two types of application interface: a Web-based interface and an android application interface. We have implemented a real-time weather observation system embedded the proposed application framework. To evaluate the implemented system, which is embedded the proposed application, actual crawling geotagged tweets posted on the Twitter site were used. We observed real time weather topics and our system could successfully detect bursty areas of observed emergency topics that is related to weather topics. In the future work, we are planning to develop an alert system that provides the details of detected bursty areas for users located near the bursty areas. We will also extend our proposed method to support multi-languages. Moreover, to improve the usability of our system, we have to develop an automatic method for making train set because making train sets is difficult for end users.

## Additional files

Additional file 1
**The screen chapter of the weather observation system embedded with the proposed application.**

Additional file 2
**The screen chapter of Figure** 7.
